# Co-ensiling whole-plant mulberry with navel orange residue enhances fermentation quality, bacterial community, and *in vitro* digestibility

**DOI:** 10.5713/ab.250683

**Published:** 2025-12-18

**Authors:** Rongqiang Chen, Qiang Zhang, Xiang Ou, Xianhong Cao, Lijuan Wu, Hai Lian, Hualiang Xie, Xianghui Zhao, Xiaowen Lei

**Affiliations:** 1 Ganzhou Key Laboratory for the Exploration and Utilization of Novel Feed Resources, Ganzhou Animal Husbandry and Fisheries Research Institute, Gannan Academy of Sciences, Ganzhou, China; 2 Jiangxi Province Key Laboratory of Animal Nutrition/Engineering Research Center of Feed Development, College of Animal Science and Technology, Jiangxi Agricultural University, Nanchang, China

**Keywords:** Bacterial Community, Fermentation Quality, *In Vitro* Digestibility, Mixing Ratio, Navel Orange Residue, Whole-plant Mulberry

## Abstract

**Objective:**

Navel orange residue (NOR) is considered to improve the silage quality of whole-plant mulberry (WPM) because of its high water-soluble carbohydrate (WSC) content. In order to investigate the mechanism underlying NOR regulated WPM silage quality, the chemical composition, fermentation quality, bacterial community, and *in vitro* digestibility of WPM and NOR mixed silages were analyzed.

**Methods:**

WPM and NOR were mixed at ratios of 10:0 (MCK), 7:3 (M7O3), 5:5 (M5O5), and 3:7 (M3O7) on a dry matter (DM) basis. Samples were collected after 15, 30, and 45 days of ensiling to study their chemical composition, fermentation quality, bacterial community, and *in vitro* digestibility. The optimal treatment was identified through membership function analysis.

**Results:**

In comparison with MCK, incorporating 30%–70% NOR ensiled for 15–45 days significantly increased DM, WSC, lactic acid, and acetic acid (AA) contents (p<0.05). At the same time, it resulted in a significant decrease in the levels of crude protein, neutral detergent fiber, acid detergent fiber, pH, and NH_3_-N (p<0.05). The NOR addition encouraged the beneficial heterofermentative species *Lactobacillus pontis*, *Lactobacillus panis*, and *Lactobacillus buchneri*, whilst preventing unfavorable microorganisms (p<0.05). In addition, *in vitro* rumen fermentation analysis demonstrated that adding 30%–70% NOR and ensiling for 15–30 days markedly increased *in vitro* DM digestibility, gas production, total volatile fatty acids, AA, and propionic acid (PA) (p<0.05), along with a significant decrease in the AA/PA ratio (p<0.05). M3O7 ensiled for 15 days and M5O5 ensiled for 30 days achieved high membership function values of 0.839 and 0.732, respectively.

**Conclusion:**

Co-ensiling WPM with 30%–70% NOR for 15–45 days significantly enhanced fermentation quality, improved bacterial diversity, and increased *in vitro* digestibility. Overall, the optimal strategies for producing high-quality silage are co-ensiling WPM with either 70% NOR for 15 days or 50% NOR for 30 days.

## INTRODUCTION

With the rapid development of China’s ruminant industry, the demand for protein-rich forage is steadily increasing. A major limitation for the growth of ruminants farming is local low availability of high protein forage resources like alfalfa, oats, etc. The deficiency of protein forage needs to be solved through the utilization of innovative protein feed resource.

Mulberry (*Morus alba* L.), belonging to the Moraceae family, is a perennial, deep-rooted woody plant. It adjusts to different environments like mountains, grasslands, irrigated fields, degraded lands and polluted areas [1]. This plant yields significant biomass output each year, with leaf production of 25–30 tons per hectare [2]. Mulberry leaves are rich in protein, comprising 15% to 25% of the dry matter (DM). Mulberry leaves also contains bioactive compounds including polyphenols, flavonoids, alkaloids, and polysaccharides [3]. They exhibit a high dry matter digestibility with 75% to 85% [2]. Previous reports have shown that incorporating mulberry leaves into the diets of ruminants can improve rumen fermentation and ruminants’ productivity [4,5]. Overall, mulberry can be a good protein supplement for ruminant feeding due to its high biomass yield, rich nutrient profile, and superior digestibility.

The adoption of high-density farming allows for the mechanical harvesting of whole-plant mulberry (WPM) from dwarfed trees, replacing the conventional practice of manual leaf harvesting. However, the high moisture content of fresh WPM creates a long-term storage problem. Silage is an economical method to maximize the preservation of nutrients in forage. Because of its low water-soluble carbohydrate (WSC) content and high buffering capacity, the ensiling of WPM alone is difficult [6]. Yang et al [7] discovered that co-ensiling mulberry leaves with 20% smooth bromegrass (a grass containing 6%–12% WSC) for 60 days improved fermentation quality. Similarly, Wang et al [8] demonstrated that co-ensiling *Sesbania cannabina* (an annual legume containing 18.10% crude protein [CP]) with 30%–70% sweet sorghum (rich in WSC) for 60 days enhanced silage quality. Previous research suggests that co-ensiling WPM with high-WSC materials may similarly improve its silage quality.

As one of the largest global producers of oranges, China yielded approximately 7.76 million metric tons of oranges in 2023 [9]. The navel orange is a significant variety cultivated in Ganzhou. Navel orange juice processing produces a large amount of navel orange residue (NOR), which constitutes approximately 45%–60% of the raw fruit weight [10]. Currently, most NOR is discarded, leading to resource waste and pollution. NOR contains WSC and bioactive compounds which include organic acids, dietary fiber, essential oils, pectin, pigment, and flavonoids [11]. Previous studies have demonstrated that orange byproducts can be alternative energy feedstuff that can replace part of the corn in the diet of cattle or sheep [12,13]. Nonetheless, due to its high moisture content, storing fresh NOR for a long time is tough. It limits its large-scale application in ruminant feed.

Considering the complementary nutritional profiles of WPM and NOR, we propose that co-ensiling WPM with NOR may enhance silage quality. However, further research is needed to determine the optimal inclusion rate of NOR and the duration of ensiling. Therefore, the present study aimed to assess the effect of ensiling mixtures of WPM and NOR under different mixing ratios on the chemical composition, fermentation quality, bacterial community, and *in vitro* digestibility during the 15–45 days ensiling period.

## MATERIALS AND METHODS

### Raw materials and silage preparation

**Figure 1:**
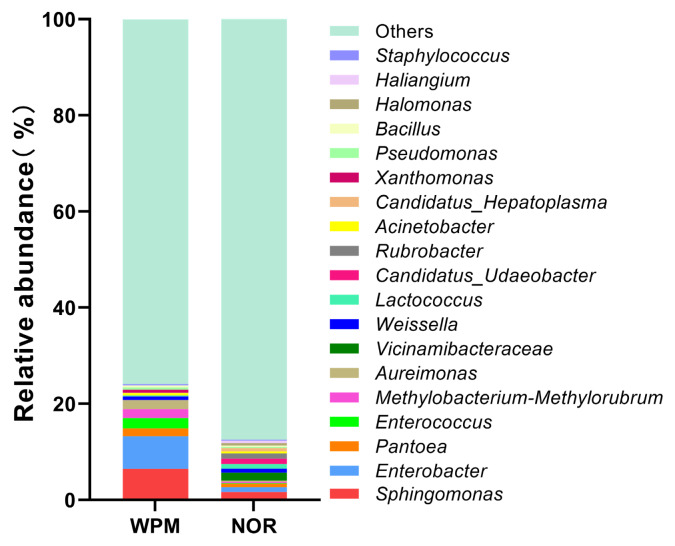
Relative abundance of major bacterial genera in WPM and NOR before ensiling. WPM, whole-plant mulberry; NOR, navel orange residue.

The WPM of Yuesang 11 cultivar was procured from Dingrui Animal Husbandry. Plants with a height of 110–130 cm were cut with sickles, leaving 10–20 cm of stubble. After harvesting, the WPM was moderately dried and cut into small segments of 2–3 cm. The NOR was sourced from Xinfeng Nongfu Spring Fruit Industry and processed by mechanical pressing to achieve a moisture content of approximately 65%. WPM and NOR were mixed at ratios of 10:0 (MCK treatment), 7:3 (M7O3 treatment), 5:5 (M5O5 treatment), and 3:7 (M3O7 treatment) based on the DM content. The natural lactic acid bacteria (LAB) populations on both WPM and NOR raw materials were found to be insufficient, with a relative abundance of less than 2% (Figure 1). To ensure a rapid and dominant fermentation by desirable LAB, each treatment was inoculated with *Lactobacillus plantarum* (Shandong Hezong Kangyuan Biotechnology) and *Limosilactobacillus fermentum* (Shandong Hezong Kangyuan Biotechnology) at 1.0×10^6^ and 5.0×10^5^ colony-forming units (CFU) per gram of fresh weight (FW), respectively. The moisture content of each treatment was first measured rapidly using a moisture analyzer (TSF-10A; Shanghai Yoke Instrument & Meter). The required amount of sterilized water to achieve a moisture content of approximately 65% was calculated based on initial measurements. After the required amount of sterilized water was added to each treatment, the materials were thoroughly mixed. After that, the materials (about 1,000 g) were immediately packed into plastic silo bags (23×40; Wenzhou Wangting Packaging) and tightly vacuum-sealed using a vacuum sealer (SF001; Zhejiang SUPOR). The 36 silage bags (4 treatments×3 ensiling days×3 replicates) were opened to determine the chemical composition, fermentation quality, bacteria communities, and *in vitro* digestibility after 15, 30, and 45 days of storage at room temperature (25±5°C). Characteristics of WPM and NOR before ensiling are presented in Table 1.

### Chemical and fermentation profile analyses

Samples from each treatment were divided into four subsamples after 15, 30, and 45 days of ensiling. The first subsample, weighing approximately 200 g, was dried at 65°C for 48 h to determine the DM content. Then, the dried samples were ground to pass through 1-mm screen using a laboratory knife mill (FW100; Taisite Instruments) for later analysis. Total nitrogen (TN) content was determined using the Kjeldahl method. The CP content was calculated by multiplying TN by 6.25. Neutral detergent fiber (NDF) and acid detergent fiber (ADF) contents were measured according to the Van Soest method. WSC content was analyzed according to the anthrone method.

For the second subsample, 20 g was mixed with 180 mL of distilled water for one minute and then filtered through four layers of cheesecloth. The pH of the filtrate was immediately measured using a glass electrode pH meter (PHS–3C; INESA Scientific Instrument). Lactic acid (LA), acetic acid (AA), propionic acid (PA), and butyric acid (BA) were quantified via high-performance liquid chromatography, as described by Xie et al [14]. The concentration of ammonia nitrogen (NH_3_-N) was determined using the sodium hypochlorite and phenol method.

### Bacterial community analysis

According to the methodology described by Zhang et al [15], the total DNA of the microbial community in the third silage subsample was extracted using a E.Z.N.A. soil DNA Kit (Omega Bio-Tek). The extracted DNA was subjected to triplicate PCR amplification using a Q5 High-Fidelity DNA Polymerase System (New England Biolabs), targeting the V3–V4 region of the 16S rRNA gene with primers 341F (5′-CCTACG GGNGGCWGCAG-3′) and 806R (5′-GGACTACHVGGGT WTCTAAT-3′). After purification and quantitation, the PCR products were sequenced using the Illumina MiSeq platform (Illumina). The resulting sequences were analyzed using the QIIME 2.0 software (University of Colorado). Bacterial alpha diversity was assessed using the Ace, Chao1, Shannon, and Simpson indices.

### *In vitro* incubation and degradability measurement

The *in vitro* experiment was conducted according to Menke’s protocol [16]. The rumen fluid was collected from four healthy Simmental crossbred steers (600 ± 20 kg body weight) prior to morning feeding. The collected fluid was filtered through four layers of gauze into a preheated (39°C) CO_2_-flushed insulated container and promptly transported to the laboratory. Filtered rumen fluid was mixed with a buffer solution at a 1:2 ratio to prepare an artificial rumen culture medium with continuous CO_2_ infusion to maintain anaerobic conditions. 0.5 g of DM from the fourth subsample and 60 mL artificial rumen culture medium transferred into 250 mL glass bottles. Blank bottles containing only 60 mL artificial rumen culture medium without substrate were incubated simultaneously, with three blank bottles per treatment. Prior to sealing, CO_2_ was introduced into each bottle to displace oxygen, after which the bottles were immediately closed with rubber stoppers and placed in an incubator, which was conducted under the steady temperature of 39°C and fluctuating frequency of 120 r/min. Three replicates were prepared for each treatment. Gas production (GP) was measured at 2, 4, 6, 8, 10, 12, 18, 24, and 48 h using a tube marked with scale value.

After 48 h of incubation, the bottles were swirled in ice for 5 min to terminate the incubation. The pH was then measured immediately. The entire fermentation broth from each bottle was transferred to 50 mL centrifuge tubes. The broth was centrifuged at 12,000×g for 15 min at 4°C to remove impurities. The resulting filtrate was frozen and stored in a −20°C refrigerator for the determination of NH_3_-N and VFA levels. The residual material was dried at 65°C for 48 h to assess the IVDMD.

### Comprehensive analysis on the quality of mixed silage

The quality of mixed silage was evaluated using membership function analysis. The samples were comprehensively ranked based on the mean membership function values, where a higher value indicates a superior silage quality. The DM, CP, WSC, LA, IVDMD, GP, total volatile fatty acids (tVFA) content are positively correlated with silage quality. The membership function value is computed using the following formula:


(1) $$U\left(X_{i}\right)=\frac{X_{i}-X_{i\;min}}{X_{i\;max}-X_{i\;min}}$$

The NDF, ADF, pH, AA, PA, NH_3_-N content are negatively correlated with silage quality. The membership function value calculation formula is as follows:


(2) $$U\left(X_{i}\right)=1-\frac{X_{i}-X_{i\;min}}{X_{i\;max}-X_{i\;min}}$$

In the equation, *U*(*X_i_*) denotes the membership function value of a certain measured index; *X_i_* represents the measured value of that index; *X_max_* represents the maximum value of that index; and *X_min_* represents the minimum value of that index.

### Statistical analyses

The experimental data were analyzed using one-way or two-way ANOVA in SPSS ver. 19.0 (IBM). Duncan’s multiple range test was employed to assess significant differences across treatments and ensiling days. Pearson correlation analysis was conducted to evaluate the relationships between bacterial community and fermentation parameters. Statistical significance was defined as p<0.05. All figures were generated using GraphPad Prism 9.

## RESULTS

### Chemical and fermentation characteristics of whole-plant mulberry-navel orange residue mixed silage

The changes in the chemical composition of the WPM-NOR mixed silages are presented in Table 2. The mixing ratio significantly impacted DM, CP, NDF, ADF and WSC contents (p<0.01). Compared with the MCK treatment, the M7O3, M5O5, and M3O7 treatments had significantly higher contents of DM and WSC (p<0.05), while the M5O5 and M3O7 treatments showed significantly lower contents of CP, NDF, and ADF (p<0.05). As the proportion of NOR in the silage increased, the CP, NDF, and ADF contents progressively decreased, while DM and WSC contents showed a consistent upward trend. The ensiling days significantly impacted DM and WSC contents (p<0.01). At 45 days of ensiling, the MCK and M7O3 treatments exhibited a significant reduction in DM content (p<0.05) relative to day 15. Meanwhile, M7O3 treatment exhibited a significant reduction in WSC content (p<0.05).

**Figure 2:**
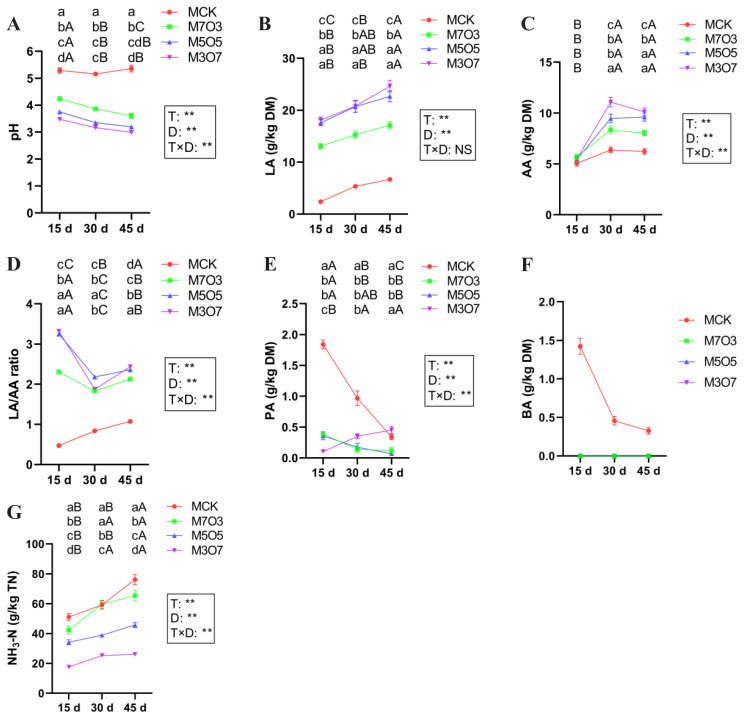
Changes in (A) pH, (B) LA, (C) AA, (D) LA/AA ratio, (E) PA, (F) BA, and (G) NH_3_-N levels in silage during the ensiling process. MCK, 100% WPM; M7O3, 70% WPM and 30% NOR; M5O5, 50% WPM and 50% NOR; M3O7, 30% WPM and 70% NOR. Bars indicate the standard error of the mean. ^A–C^ Capital letters indicate significant differences among different ensiling days within the same treatment (p<0.05). ^a–d^ Small letters represent significant differences among treatments on the same day of ensiling (p<0.05). ** p<0.01; NS, no significance. T, treatment; D, ensiling days; T×D, the interaction between treatment and ensiling days; DM, dry matter; LA, lactic acid; AA, acetic acid; PA, propionic acid; BA, butyric acid; NH_3_-N, ammonia nitrogen; TN, total nitrogen; WPM, whole-plant mulberry; NOR, navel orange residue.

The changes in the fermentation characteristics of the WPM-NOR mixed silages are presented in Figure 2. The mixing ratio had significant effects on pH, LA, AA, LA/AA ratio, PA, and NH_3_-N contents (p<0.01). The MCK treatment maintained suboptimal pH (>5.1), significantly higher (p< 0.05) than M7O3, M5O5, and M3O7 treatments (all<4.24). The LA content in M7O3, M5O5, and M3O7 treatments was significantly higher (p<0.05) than MCK treatment. The AA content in the M7O3, M5O5, and M3O7 treatments was significantly greater (p<0.05) than MCK treatment after 30 days of ensiling. The LA/AA ratios of M7O3, M5O5, and M3O7 treatments were significantly higher (p<0.05) than MCK treatment. Moreover, all LA/AA ratios remained below 3.32 throughout the ensiling period. The PA content in the M7O3 and M5O5 treatments was significantly lower (p<0.05) than MCK treatment after 15 days of ensiling. The PA content in M3O7 treatment was significantly lower (p<0.05) than MCK treatment at 15 and 30 days of ensiling. BA was only present in the MCK treatment. Furthermore, the NH_3_-N content in the M5O5 and M3O7 treatments was significantly lower (p<0.05) than that in the MCK and M7O3 treatments after 15 days of ensiling. The NH_3_-N content in the M7O3 treatment was notably lower (p<0.05) than that in the MCK treatment at 15 and 45 days of ensiling. The ensiling days had significant effects on pH, LA, AA, LA/AA ratio, PA, and NH_3_-N contents (p<0.01). As the ensiling period extended from 15 to 45 days, the pH in the MCK treatment remained stable, while a declining trend was observed in the M7O3, M5O5, and M3O7 treatments. The contents of LA and NH_3_-N showed an increasing trend across all treatments. In contrast, AA content initially increased, peaked at 30 days, and then decreased slightly. The PA content exhibited a decreasing trend in the MCK, M7O3, and M5O5 treatments, whereas an increasing trend was noted in the M3O7 treatment. The interaction between mixing ratio and ensiling days had a significant effect on pH, AA, the LA/AA ratio, PA, and NH_3_-N contents (p< 0.01). Overall, co-ensiling WPM with 30%–70% NOR for 15–45 days improved fermentation quality, with increasing NOR proportions progressively lowering pH and NH_3_-N content while boosting LA and AA production.

### Temporal dynamics of bacterial communities in whole-plant mulberry-navel orange residue mixed silage

The alpha diversity analysis of the WPM-NOR mixed silages are presented in Table 3. The mixing ratio had significant effects on Ace, Chao1, Shannon, and Simpson indices (p<0.01). At 15 days of ensiling, the Ace and Chao1 indices of the M3O7 treatment were significantly lower (p<0.05) than those of the other treatments. At 30 and 45 days of ensiling, the Ace and Chao1 indices of the M7O3, M5O5, and M3O7 treatments were significantly lower (p<0.05) than those of the MCK treatment, with the M3O7 treatment showing the lowest values. The Shannon index of the M7O3, M5O5, and M3O7 treatments was significantly lower (p<0.05) than that of the MCK group, with the M3O7 treatment being the lowest. At 15 days of ensiling, the Simpson index of the M5O5 and M3O7 treatments was significantly lower (p<0.05) than that of the MCK treatment. At 30 and 45 days of ensiling, the Simpson index of the M7O3, M5O5, and M3O7 treatments was significantly lower (p<0.05) than that of the MCK treatment, with the M3O7 treatment being the lowest. Notably, as the NOR inclusion rate increased, all four indices exhibited a progressive decline, indicating reduced bacterial community alpha diversity. The ensiling days had significant effects on Ace, Chao1, Shannon, and Simpson indices (p<0.01). As the ensiling period extended from 15 to 45 days, the Ace and Chao1 indices in the MCK and M7O3 treatments showed a significant declining trend (p<0.05). Meanwhile, these indices in the M5O5 and M3O7 treatments, along with the Shannon index in M3O7 and the Simpson index in M7O3, initially decreased significantly (p<0.05), reached their lowest point at day 30, and then increased slightly. The interaction between mixing ratio and ensiling days had a significant effecton Ace, Chao1, Shannon, and Simpson indices (p<0.01).

**Figure 3:**
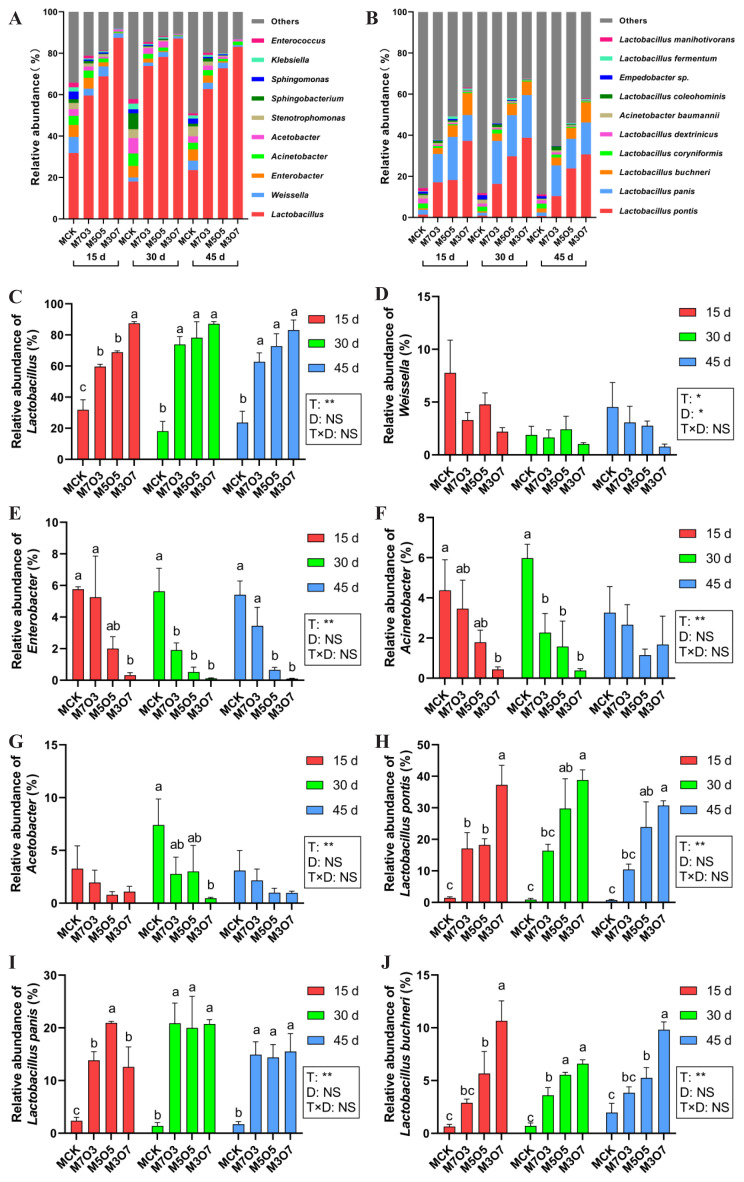
Bacterial community composition in silage during ensiling. (A) Genus-level and (B) species-level profiles; relative abundance of (C) *Lactobacillus*, (D) *Weissella*, (E) *Enterobacter*, (F) *Acinetobacter*, (G) *Acetobacter*, (H) *Lactobacillus pontis*, (I) *Lactobacillus panis*, and (J) *Lactobacillus buchneri*. MCK, 100% WPM; M7O3, 70% WPM and 30% NOR; M5O5, 50% WPM and 50% NOR; M3O7, 30% WPM and 70% NOR. Bars indicate the standard error of the mean. ^a–c^ Small letters represent significant differences among treatments on the same day of ensiling (p<0.05). * p<0.05; ** p<0.01; NS, no significance. T, treatment; D, ensiling days; T×D, the interaction between treatment and ensiling days; WPM, whole-plant mulberry; NOR, navel orange residue.

The dynamics of relative abundance among bacterial communities at the genus and species levels are shown in Figures 3A, 3B, respectively. *Lactobacillus*, *Weissella*, *Enterobacter*, *Acinetobacter*, and *Acetobacter* were the dominant genera across four treatments. The mixing ratio significantly modulated (p<0.05) the relative abundance of the five dominant genera. Ensiling days had a notable impact (p<0.05) on *Weissella*. The relative abundance of *Lactobacillus* was significantly higher (p<0.05) in the M7O3, M5O5, and M3O7 treatments than in the MCK treatment (Figure 3C), with the M3O7 treatment showing the highest value. At 15 days of ensiling, the relative abundance of *Enterobacter* in the M3O7 treatment was significantly lower than that in the MCK treatment (Figure 3E). At 30 days of ensiling, the relative abundances of *Enterobacter* in the M7O3, M5O5, and M3O7 treatments had decreased significantly (p<0.05) compared to the MCK treatment. At 45 days of ensiling, the relative abundances of *Enterobacter* in the M5O5 and M3O7 treatments remained significantly lower (p<0.05) than those in the MCK treatment. At 15 days of ensiling, the relative abundance of *Acinetobacter* was significantly reduced (p<0.05) in M3O7 treatment compared to the MCK treatment (Figure 3F). At 30 days of ensiling, the relative abundances of *Acinetobacter* in the M7O3, M5O5, and M3O7 treatments had decreased significantly (p<0.05) compared to the MCK treatment. The relative abundance of *Acetobacter* in the M3O7 treatment was significantly lower (p< 0.05) than that in the MCK treatment at 30 days of ensiling (Figure 3G). *Lactobacillus pontis*, *Lactobacillus panis*, and *Lactobacillus buchneri* were the dominant species across four treatments. The mixing ratio significantly affected (p<0.05) the relative abundance of the three dominant species. At 15 days of ensiling, the relative abundance of *L. pontis* significantly increased (p<0.05) in the M7O3, M5O5, and M3O7 treatments compared with the MCK treatment (Figure 3H). At 30 days and 45 days of ensiling, the relative abundance of *L. pontis* significantly increased (p<0.05) in the M5O5, and M3O7 treatments compared with the MCK treatment. The relative abundance of *L. panis* was significantly higher (p< 0.05) in the M7O3, M5O5, and M3O7 treatments compared with the MCK treatment (Figure 3I). At 15 days and 45 days of ensiling, the relative abundance of *L. pontis* significantly increased (p<0.05) in the M5O5, and M3O7 treatments compared with the MCK treatment (Figure 3J). At 30 days of ensiling, the relative abundance of *L. pontis* significantly increased (p<0.05) in the M7O3, M5O5, and M3O7 treatments compared with the MCK treatment. Overall, co-ensiling WPM with 30%–70% NOR for 15–45 days promoted the growth of heterofermentative *Lactobacillus* species, notably *L. pontis*, *L. panis*, and *L. buchneri*, while effectively inhibiting undesirable microorganisms, particularly *Enterobacter* and *Acinetobacter*.

**Figure 4:**
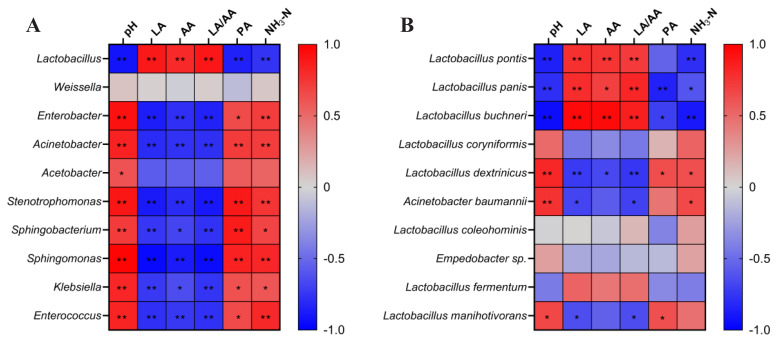
Correlation analysis between the bacterial community and silage fermentation parameters after 30 days of ensiling. (A) Genus-level correlations with fermentation parameters, (B) species-level correlations with fermentation parameters. * p<0.05; ** p<0.01. LA, lactic acid; AA, acetic acid; PA, propionic acid; NH_3_-N, ammonia nitrogen.

To gain deeper insights into the silage fermentation process, we further evaluated the correlations between fermentation characteristics and the bacterial community in WPM-NOR mixed silage after 30 days of ensiling (Figure 4). Our analysis revealed that *Lactobacillus* was a significantly negatively correlated with pH, PA, and NH_3_-N contents (p<0.01), and positively correlated with LA, AA, and LA/AA (p<0.01). *Enterobacter*, *Acinetobacter*, *Stenotrophomonas*, *Sphingobacterium*, *Sphingomonas*, *Klebsiella*, and *Enterococcus* were positively linked to pH, PA, and NH_3_-N contents (p<0.05), but negatively associated with LA, AA, and LA/AA (p<0.05). *L. pontis* was negatively correlated with pH and NH_3_-N contents (p<0.01), but showed positive associations with LA, AA, and LA/AA (p<0.01). *L. panis* and *L. buchneri* were negatively correlated with pH, PA, and NH_3_-N contents (p<0.05), but showed positive associations with LA, AA, and LA/AA (p< 0.05).

### *In vitro* digestibility of whole-plant mulberry-navel orange residue mixed silage

**Figure 5:**
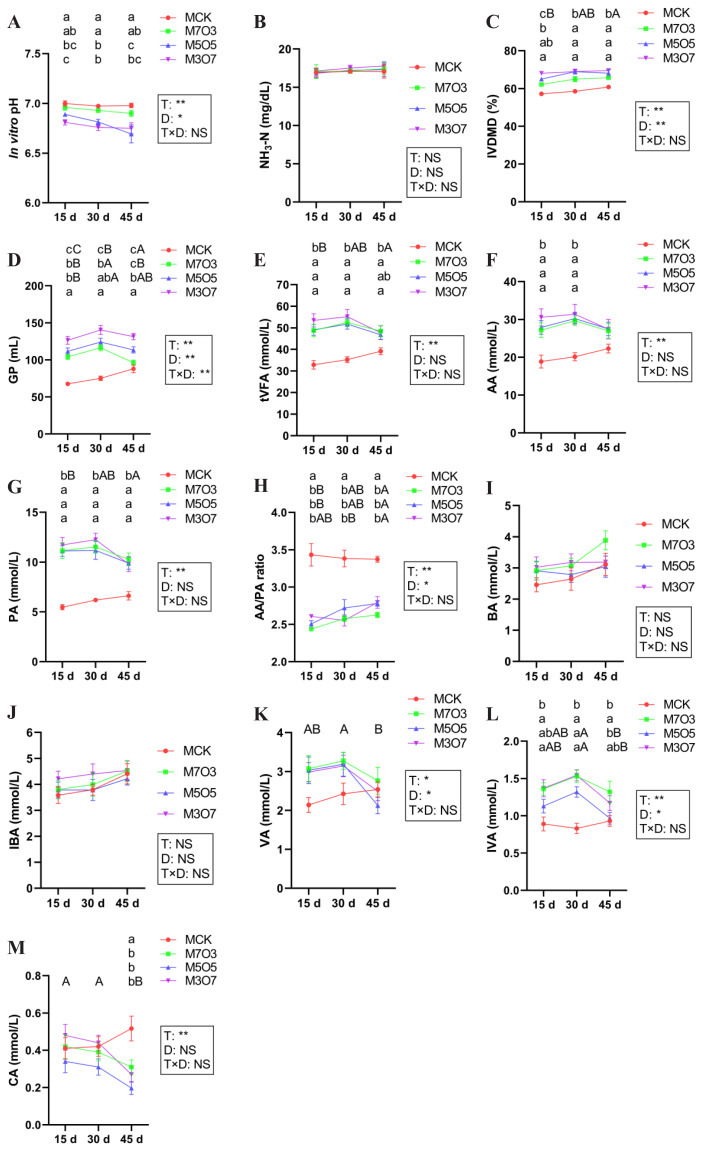
Changes in (A) *in vitro* pH, (B) NH_3_-N, (C) IVDMD, (D) GP, (E) tVFA, (F) AA, (G) PA, (H) AA/PA ratio, (I) BA, (J) IBA, (K) VA, (L) IVA, and (M) CA levels during *in vitro* fermentation (48 h) of silage after 15, 30, and 45 days of ensiling. MCK, 100% WPM; M7O3, 70% WPM and 30% NOR; M5O5, 50% WPM and 50% NOR; M3O7, 30% WPM and 70% NOR. Bars indicate the standard error of the mean. ^A,B^ Capital letters indicate significant differences among different ensiling days within the same treatment (p<0.05). ^a–c^ Small letters represent significant differences among treatments on the same day of ensiling (p<0.05). * p<0.05; ** p<0.01; NS, no significance. T, treatment; D, ensiling days; T×D, the interaction between treatment and ensiling days; IVDMD, *in vivo* dry matter digestibility; GP, gas production; tVFA, total volatile fatty acids; AA, acetic acid; PA, propionic acid; AA/PA ratio, acetic acid/propionic acid ratio; BA, butyric acid; IBA, isobutyric acid; VA, valeric acid; IVA, isovaleric acid; CA, caproic acid; WPM, whole-plant mulberry; NOR, navel orange residue.

**Figure 6:**
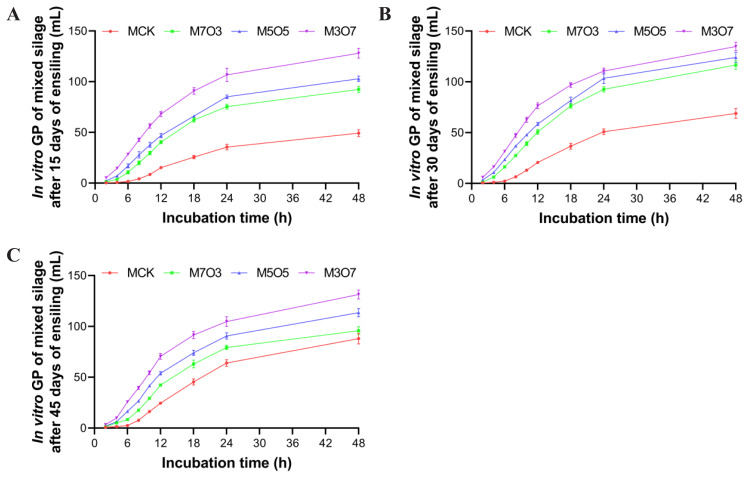
The 48 h *in vitro* GP profiles of WPM and NOR mixed silages after 15 (A), 30 (B), and 45 days (C) of ensiling. MCK, 100% WPM; M7O3, 70% WPM and 30% NOR; M5O5, 50% WPM and 50% NOR; M3O7, 30% WPM and 70% NOR. Bars indicate the standard errors of the means. GP, gas production; WPM, whole-plant mulberry; NOR, navel orange residue.

The results of *in vitro* rumen fermentation are presented in Figure 5. The mixing ratio significantly influenced the pH, IVDMD, GP, tVFA, AA, PA, AA/PA ratio, VA, IBA, and CA levels (p<0.05). Ensiling days also had a significant impact on pH, IVDMD, GP, AA/PA ratio, VA, IVA levels (p<0.05). Moreover, a significant interaction between the mixing ratio and ensiling days was observed for GP (p<0.05). Regarding *in vitro* fermentation pH, the M5O5 and M3O7 treatments exhibited significantly lower values (p<0.05) compared with the MCK treatment, and all treatments maintained pH values within the optimal range of 6.69–7.00. The IVDMD of the M7O3, M5O5, and M3O7 treatments was significantly higher (p<0.05) than that of the MCK after 30 days of ensiling, with relative improvements of 11.04%, 17.79%, and 17.86%, respectively. Furthermore, after 30 days of ensiling, cumulative GP was significantly higher (p<0.05) in the M7O3, M5O5, and M3O7 treatments than in the MCK treatment, with peak values reaching 92.69, 103.51, and 110.71 mL, respectively. This corresponded to significant increases of 69.13%, 80.05%, and 95.58% over the MCK treatment. The M7O3, M5O5, and M3O7 treatments showed significantly higher peak levels of tVFA, AA, and PA (p<0.05) compared with the MCK treatment at 30 days of ensiling, with the following quantitative improvements: tVFA levels increased by 48.91%, 46.78%, and 56.54%, respectively, and AA levels rose by 47.37%, 50.30%, and 56.01%, respectively, and PA levels demonstrated increases of 86.41%, 80.91%, and 98.22%, respectively. The AA/PA ratios in the M7O3, M5O5, and M3O7 treatments ranged between 2.44 and 2.79, all being significantly lower (p<0.05) than that in the MCK treatment. The IVA levels in M7O3, M5O5, and M3O7 treatments were significantly higher (p< 0.05) than that in the MCK treatment at 30 days of ensiling. Overall, co-ensiling WPM with 30%–70% NOR for 15–45 days significantly improved IVDMD and GP, increased tVFA, AA, and PA levels, and reduced pH and the AA/PA ratio. The dynamic profiles of GP are presented in Figure 6. The treatments exhibited progressively steeper GP curves during the initial 2–12 h with increasing NOR content. Over the 48 h incubation period, cumulative GP increased sequentially across treatments as follows: MCK<M7O3<M5O5<M3O7.

### Comprehensive analysis on the quality of whole-plant mulberry-navel orange residue mixed silage

Table 4 summarizes the membership function evaluation of 13 critical silage quality metrics for all treatments after 15, 30 and 45 days of ensiling. The average membership function values across the groups ranked as follows: D15 (0.893)>D30 (0.773)>D45 (0.769)>C30 (0.732)>C15 (0.713)>C45 (0.711)>B30 (0.612)>B15 (0.604)>B45 (0.575)>A45 (0.309)>A30 (0.267)>A15 (0.224). Notably, the M3O7 treatment exhibited its maximum membership function value after 15 days of ensiling, whereas both M5O5 and M7O3 treatments demonstrated peak values at 30 days.

## DISCUSSION

### Effects of navel orange residue on the fermentation quality of whole-plant mulberry-navel orange residue mixed silage

High-quality silage fermentation is characterized by a pH below 4.2 [17]. In this study, the MCK treatment maintained a pH above 5.1 throughout the entire ensiling period, demonstrating inadequate fermentation when WPM was ensiled alone. This finding was consistent with the results reported by Hao et al [18]. The low LA content in the MCK treatment further confirmed inadequate LAB fermentation. Notably, as the proportion of NOR increased, a significant reduction in pH and a concurrent increase in LA content were observed. This can be attributed to two key factors: the naturally low pH of NOR due to its high phenolic acid content [19], and the elevated WSC content in NOR, providing additional fermentable substrates that stimulate *Lactobacillus* activity and enhance LA production [20]. As reported in a previous study, the LA/AA ratio serves as a reliable metabolic marker, where ratio>3.0 typically indicate homofermentative dominance, while ratio<3.0 suggest heterofermentative predominance [21]. Our results showed that NOR-supplemented treatments (M7O3, M5O5, and M3O7) exhibited LA/AA ratios ranging from 1.83 to 2.43 after 30 days of ensiling, with corresponding AA concentrations significantly elevated compared with those in the MCK treatment. The findings suggested that the inclusion of NOR selectively modulates heterofermentative pathway activity in LAB fermentation, primarily driven by heterofermentative LAB such as *L. pontis*, *L. panis* and *L. buchneri*. BA, an undesirable metabolite produced by bacteria such as *Clostridium*, is a critical negative indicator of silage quality [22]. In this study, BA was exclusively detected in the MCK treatment, which could be attributed to *Clostridium tyrobutyricum* proliferation during fermentation. NH_3_-N levels indicate the degree of protein and amino acid breakdown during ensiling, largely due to plant proteases and the metabolic activity of microbes such as *Clostridium* and *Enterobacter* [23,24]. In this study, the significantly lower NH_3_-N content in NOR-supplemented silages compared with the WPM silage, along with the strongly positive correlation between *Enterobacter* and NH_3_-N and the strongly negative correlation between *Enterobacter* and pH, suggested that NOR addition possibly suppresses *Enterobacter* and *Clostridium* by promoting a rapid pH drop.

### Bacterial community shifts in response to navel orange residue amendment in whole-plant mulberry-navel orange residue mixed silage

Decreases in the Ace, Chao1, Shannon, and Simpson indices often indicate successful ensiling because of the increased dominance of beneficial LAB over other microorganisms [25]. In this study, increasing NOR levels corresponded with reduced bacterial alpha diversity, indicating a positive effect of NOR on silage fermentation.

NOR is rich in WSC, with its fructose content being significantly higher than its glucose content [26]. Heterofermentative LAB species possess a competitive advantage over homofermentative LAB in utilizing fructose [27]. Wang et al [8] demonstrated that co-ensiling *Sesbania cannabina* with 30–70% sweet sorghum improved bacterial diversity. This was evidenced by an initial dominance of homofermentative species *Lactobacillus plantarum* and *Lactobacillus farciminis* during early fermentation stages (0–7 days), followed by an ecological succession to heterofermentative species *L. buchneri* and *Lactobacillus hilgardii* in later phases (14–60 days). Consistent with Wang et al’s findings, our results demonstrate that incorporation of 30%–70% NOR promoted beneficial heterofermentative LAB species, specifically *L. pontis*, *L. panis*, and *L. buchneri* throughout the 15–45 days ensiling period. Therefore, we postulate that the addition of NOR drives a dynamic bacterial succession mediated by substrate availability. The abundant WSC (particularly fructose) in NOR provides a substrate basis for the rapid proliferation of LAB. In the early ensiling stage (0–15 days), both glucose and fructose are rapidly co-utilized by various LAB. As fermentation progresses into the later stage (15–45 days), the preferential depletion of glucose creates a microenvironment enriched in residual fructose, which in turn exerts a selective pressure. This favors the ecological dominance of heterofermentative LAB (such as *L. pontis*, *L. panis*, and *L. buchneri*) that are adept at utilizing fructose. The metabolic activity of these dominant heterofermenters results in significant accumulation of LA and AA, synergistically establishing a low pH environment that effectively inhibits harmful microorganisms such as *Enterobacter* and *Clostridium*. This targeted microbial suppression leads to a simplified community structure, which is directly reflected in the observed decrease in bacterial alpha diversity. Consequently, this microbial optimization not only reduces protein degradation (as indicated by lower NH_3_-N) but also minimizes DM losses from undesirable microbial respiration, thereby comprehensively enhancing both the fermentation quality and nutritional preservation of the silage.

### Effects of navel orange residue on the *in vitro* digestibility of whole-plant mulberry-navel orange residue mixed silage

The pH of ruminal fluid is a critical parameter for assessing rumen fermentation dynamics [28]. In this study, the mean ruminal fluid pH ranged from 6.69 to 7.00, which aligned with the optimal range (6.0–7.0) for bacterial activity and fiber digestion [29]. Zhang et al [30] demonstrated that increasing the proportion of whole-crop corn in alfalfa silage significantly reduced *in vitro* pH, while concurrently increasing IVDMD and GP contents. Our findings showed a comparable trend, with increasing NOR inclusion ratios in WPM silage leading to progressively lower *in vitro* pH. The observed pH reduction may be attributed to two primary factors: the inherently acidic properties of NOR-enriched silage matrices, and the substantial accumulation of tVFA during *in vitro* ruminal fermentation [31]. The NH_3_-N concentration serves as a direct indicator of the equilibrium between proteolytic degradation and microbial assimilation rates [32]. In our study, NH_3_-N concentrations of four treatments ranged from 16.82 to 17.80 mg/dL, which were within the range (6.30–27.50 mg/dL) considered optimal for rumen microbial growth [33]. Higher DM digestibility values reflect enhanced feed degradability and greater nutrient utilization in ruminants. In this study, IVDMD increased progressively with increasing proportions of NOR, mirroring the findings reported by Zhang et al [30]. GP, a key biomarker of rumen microbial activity, has kinetics primarily determined by the nutritional characteristics of feed substrates. Rapidly fermentable components, such as starch and WSC, promote increased GP. In contrast, structural carbohydrates (NDF and ADF) exhibit slower degradation kinetics, leading to reduced GP [34]. In this study, the treatments with higher NOR proportions exhibited a steeper GP curve in the initial 2–12 h, along with higher cumulative GP for 48 h. This could be attributed to the higher NOR proportion in the mixed silage, which provided abundant WSC to stimulate rapid fermentation initiation and further enhanced more comprehensive substrate utilization by the rumen microbial community. VFAs, predominantly AA, PA, and BA, collectively provide approximately 75% of the metabolizable energy requirements for ruminants [35]. Acetate is the principal precursor for lipogenesis in both mammary and adipose tissues, while propionate acts as the major gluconeogenic substrate. A reduced AA/PA ratio enhances dietary energy utilization efficiency in ruminants [36]. In this study, NOR-supplemented treatments exhibited significantly elevated AA and PA concentrations concomitant with a reduced AA/PA ratio. The results indicated that NOR supplementation can improve *in vitro* digestibility, likely by modulating rumen fermentation to favor PA production. Our results are similar to those of Chen et al [37], where a higher sweet sorghum ratio in alfalfa silage resulted in higher PA content and a lower AA/PA ratio.

### Comprehensive analysis on the quality of whole-plant mulberry-navel orange residue mixed silage

To overcome the limitations of single-metric evaluations in silage quality assessment, this study employed a comprehensive membership function analysis of 13 key parameters to provide a more precise and reliable method [38]. Membership function analysis revealed that NOR supplementation consistently outperformed the MCK treatment across ensiling periods. Li et al [39] found that the optimal ensiling duration varied with the mixing ratios in red clover-alfalfa mixtures. Similarly in this study, the optimal comprehensive quality was achieved at 45, 30, 30, and 15 days for the MCK, M7O3, M5O5, and M3O7 treatments, respectively. These findings can be explained by the fact that the treatments with higher NOR content were characterized by elevated WSC content, reduced buffering capacity, and a lower initial pH. These properties accelerated the fermentation process, thereby shortening the time required to achieve optimal quality.

## CONCLUSION

WPM alone exhibited poor fermentation quality during the 15–45 days ensiling period. However, the incorporation of 30%–70% NOR enhanced the silage quality of WPM. Notably, the addition of 50% or 70% NOR outperformed other treatments, resulting in higher levels of LA and AA, along with reduced pH and NH_3_-N contents. The inclusion of 50% or 70% NOR decreased bacterial alpha diversity, primarily by promoting beneficial heterofermentative *Lactobacillus* species, specifically *L. pontis*, *L. panis*, and *L. buchneri*, while inhibiting undesirable microorganisms such as *Enterobacter* and *Acinetobacter*. Besides, the inclusion of 50% or 70% NOR considerably improved *in vitro* digestibility, evidenced by an increase in IVDMD, GP and tVFA contents. Overall, the optimal strategies for producing high-quality silage are co-ensiling WPM with either 70% NOR for 15 days or 50% NOR for 30 days.

## Figures and Tables

**Table 1:** Characteristics of WPM and NOR before ensiling

Items	WPM	NOR
pH	6.23±0.04	4.97±0.03
DM (g/kg FW)	393.11±5.25	353.61±7.25
CP (g/kg DM)	155.51±8.28	74.71±2.49
NDF (g/kg DM)	426.59±8.92	213.35±10.63
ADF (g/kg DM)	327.67±7.35	194.59±7.17
WSC (g/kg DM)	62.25±2.45	256.28±16.73

WPM, whole-plant mulberry; NOR, navel orange residue; DM, dry matter; FW, fresh weight; CP, crude protein; NDF, neutral detergent fiber; ADF, acid detergent fiber; WSC, water soluble carbohydrate.

**Table 2:** Chemical composition of WPM-NOR mixed silages

Items	Treatments (T)^1)^	Ensiling days (D)			SEM	p-value^2)^		
	
		15	30	45		T	D	T×D
DM (g/kg FW)	MCK	291.61^Ab^	272.75^Bb^	261.32^Bc^	5.29	^**^	^**^	NS
	M7O3	326.27^Aa^	313.33^Aa^	292.68^Bb^				
	M5O5	324.42^a^	317.55^a^	310.43^a^				
	M3O7	333.43^a^	324.69^a^	320.75^a^				
CP (g/kg DM)	MCK	154.07^a^	149.87^a^	142.54^a^	5.27	^**^	NS	NS
	M7O3	140.95^ab^	138.15^ab^	135.66^ab^				
	M5O5	128.14^b^	125.59^b^	123.23^b^				
	M3O7	104.39^c^	100.66^c^	98.10^c^				
NDF (g/kg DM)	MCK	397.81^a^	373.66^a^	368.73^a^	15.16	^**^	NS	NS
	M7O3	337.38^b^	328.95^a^	313.32^ab^				
	M5O5	295.60^bc^	281.08^b^	264.34^bc^				
	M3O7	251.80^c^	231.81^c^	226.00^c^				
ADF (g/kg DM)	MCK	315.70^a^	310.72^a^	305.50^a^	14.12	^**^	NS	NS
	M7O3	272.41^ab^	265.87^ab^	258.71^b^				
	M5O5	251.43^b^	239.31^b^	221.39^bc^				
	M3O7	230.35^b^	221.73^c^	210.23^c^				
WSC (g/kg DM)	MCK	33.83^c^	29.38^c^	25.71^c^	7.32	^**^	^**^	NS
	M7O3	94.51^Ab^	79.79^ABb^	67.36^Bb^				
	M5O5	123.59^a^	104.35^a^	97.82^a^				
	M3O7	146.55^a^	128.24^a^	116.55^a^				

1) MCK, 100% WPM; M7O3, 70% WPM and 30% NOR; M5O5, 50% WPM and 50% NOR; M3O7, 30% WPM and 70% NOR.

2) T, treatment; D, days of ensiling; T×D, the interaction between treatment and days of ensiling.

* p<0.05;

** p<0.01; NS, no significance.

A,B Means in the same row with different capital letters are significantly different (p<0.05).

a–c Means in the same column with different small letters are significantly different (p<0.05).

WPM, whole-plant mulberry; NOR, navel orange residue; SEM, standard error of the mean; DM, dry matter; FW, fresh weight; CP, crude protein; NDF, neutral detergent fiber; ADF, acid detergent fiber; WSC, water soluble carbohydrate.

**Table 3:** Bacterial community alpha diversity in WPM-NOR mixed silage

Indexs	Treatments (T)^1)^	Ensiling days (D)			SEM	p-value^2)^		
	
		15	30	45		T	D	T×D
Ace	MCK	1,019.79^Aa^	755.76^ABa^	621.21^Ba^	73.60	^**^	^**^	^**^
	M7O3	755.23^Aa^	412.95^Bb^	393.23^Bb^				
	M5O5	961.37^Aa^	260.64^Bb^	298.64^Bbc^				
	M3O7	366.32^Ab^	160.96^Bb^	217.50^ABc^				
Chao1	MCK	1,011.53^Aa^	750.79^ABa^	616.92^Ba^	70.82	^**^	^**^	^**^
	M7O3	747.20^Aa^	410.61^Bb^	392.70^Bb^				
	M5O5	926.40^Aa^	259.15^Bb^	296.12^Bbc^				
	M3O7	355.63^Ab^	160.69^Bb^	215.20^Bc^				
Shannon	MCK	7.39^a^	7.57^a^	6.88^a^	0.24	^**^	^**^	^**^
	M7O3	6.41^b^	5.83^b^	6.12^b^				
	M5O5	6.16^b^	5.14^bc^	5.30^c^				
	M3O7	5.00^Ac^	4.56^Bc^	4.90^ABc^				
Simpson	MCK	0.98^a^	0.99^a^	0.98^a^	0.03	^**^	^**^	^**^
	M7O3	0.96^Aab^	0.94^Bb^	0.96^ABb^				
	M5O5	0.95^b^	0.92^b^	0.94^bc^				
	M3O7	0.92^c^	0.91^b^	0.93^c^				

1) MCK, 100% WPM; M7O3, 70% WPM and 30% NOR; M5O5, 50% WPM and 50% NOR; M3O7, 30% WPM and 70% NOR.

2) T, treatment; D, days of ensiling; T×D, the interaction between treatment and days of ensiling.

* p<0.05;

** p<0.01; NS, no significance.

A,B Means in the same row with different capital letters are significantly different (p<0.05).

a–c Means in the same column with different small letters are significantly different (p<0.05).

WPM, whole-plant mulberry; NOR, navel orange residue; SEM, standard error of the mean.

**Table 4:** Comprehensive analysis on the quality of WPM-NOR mixed silages

Item	A15	B15	C15	D15	A30	B30	C30	D30	A45	B45	C45	D45
DM	0.420	0.901	0.875	1.000	0.159	0.721	0.780	0.879	0.000	0.435	0.681	0.824
CP	1.000	0.766	0.537	0.112	0.925	0.716	0.491	0.046	0.794	0.671	0.449	0.000
NDF	0.000	0.352	0.595	0.850	0.141	0.401	0.679	0.966	0.169	0.492	0.777	1.000
ADF	0.000	0.410	0.609	0.809	0.047	0.472	0.724	0.891	0.097	0.540	0.894	1.000
WSC	0.067	0.569	0.810	1.000	0.030	0.448	0.651	0.848	0.000	0.345	0.597	0.752
pH	0.000	0.473	0.679	0.797	0.030	0.633	0.848	0.928	0.084	0.738	0.911	1.000
LA	0.000	0.480	0.683	0.706	0.132	0.580	0.822	0.826	0.193	0.661	0.913	1.000
AA	1.000	0.895	0.940	0.934	0.782	0.452	0.267	0.000	0.806	0.505	0.244	0.158
PA	0.000	0.819	0.836	0.983	0.492	0.960	0.944	0.842	0.847	0.972	1.000	0.785
NH_3_-N	0.428	0.576	0.716	1.000	0.289	0.281	0.635	0.867	0.000	0.184	0.518	0.854
IVDMD	0.000	0.397	0.633	0.877	0.106	0.630	0.951	0.954	0.294	0.690	0.885	1.000
GP	0.000	0.504	0.626	0.922	0.229	0.787	0.875	1.000	0.453	0.544	0.752	0.961
tVFA	0.000	0.713	0.724	0.921	0.107	0.879	0.846	1.000	0.280	0.700	0.623	0.666
Mean	0.224	0.604	0.713	0.839	0.267	0.612	0.732	0.773	0.309	0.575	0.711	0.769
Ranking	12	8	5	1	11	7	4	2	10	9	6	3

A15, MCK treatment after 15 days of ensiling; B15, M7O3 treatment after 15 days of ensiling; C15, M5O5 treatment after 15 days of ensiling; D15, M3O7 treatment after 15 days of ensiling; A30, MCK treatment after 30 days of ensiling; B30, M7O3 treatment after 30 days of ensiling; C30, M5O5 treatment after 30 days of ensiling; D30, M3O7 treatment after 30 days of ensiling; A45, MCK treatment after 45 days of ensiling; B45, M7O3 treatment after 45 days of ensiling; C45, M5O5 treatment after 45 days of ensiling; D45, M3O7 treatment after 45 days of ensiling.

WPM, whole-plant mulberry; NOR, navel orange residue; DM, dry matter; CP, crude protein; NDF, neutral detergent fiber; ADF, acid detergent fiber; WSC, water-soluble carbohydrate; LA, lactic acid; AA, acetic acid; PA, propionic acid; IVDMD, *in vitro* dry matter digestibility; GP, gas production; tVFA, total volatile fatty acids.

## Data Availability

Upon reasonable request, the datasets of this study can be available from the corresponding author.
